# Development and Validation of a Model to Predict the Contract Service of Family Doctor: A National Survey in China

**DOI:** 10.3389/fpubh.2022.750722

**Published:** 2022-04-25

**Authors:** Zhiqiang Nie, Chen Chen, Guo Chen, Chao Wang, Yong Gan, Yingqing Feng, Zuxun Lu

**Affiliations:** ^1^Department of Social Medicine and Health Management, School of Public Health, Tongji Medical College, Huazhong University of Science and Technology, Wuhan, China; ^2^Department of Cardiology, Hypertension Research Laboratory, Guangdong Cardiovascular Institute, Guangdong Provincial People's Hospital, Guangdong Academy of Medical Sciences, Guangzhou, China; ^3^Department of Respiratory, Pediatric Intensive Care Unit, Guangzhou Women and Children's Medical Center, Guangzhou Medical University, Guangzhou, China

**Keywords:** nomogram, family doctor contract service, utility, risk factors, prediction model

## Abstract

**Background:**

Previous studies have reported a relatively low utilization of family doctor contract services (FDCS) in China, while the associated factors are unknown. The current study aimed to explore the factors associated with the utilization of FDCS, and then developed and validated a predictive model based on these identified factors.

**Methods:**

We conducted a nationwide cross-sectional study using an online questionnaire, from March 2019 to April of 2019. Routinely collected variables in daily practice by family doctors were used to develop a derivation model to determine the factors associated with FDCS utilization, and then the external performance of the model was tested.

**Results:**

A total of 115,717 and 49,593 participants were included in the development and validation datasets, respectively. Nearly 6.8% of the participants who signed a contract with FDCS received healthcare services from family doctors in China. Factors associated with the utilization of FDCS included age, male sex, self-reported household income, education attainment, insurance status, self-reported health status, smoking, drinking, self-reported physical activity status, chronic disease, walking distance from the nearest community center, and illness in the last 2 weeks, with an area under the receiver operating characteristic curve (AUC) of 0.660 [95% confidence interval (CI), 0.653–0.667] and good calibration. Application of this nomogram in the validation dataset also showed acceptable diagnostic value with an AUC of 0.659 (95% CI, 0.649–0.669) and good calibration.

**Conclusion:**

Twelve easily obtainable factors in daily practice of family doctors were used to develop a model to predict the utilization of FDCS, with a moderate performance.

## Introduction

The family doctor contract service (FDCS), a core component of primary healthcare system, was expected to open a new prospect for a tiered medical system in China (1). FDCS system provides a proactive, consistent, comprehensive, stable, and affordable service to the general population by establishing a stable connection between family doctors and community residents.

The FDCS was first launched in China in 2009 as an innovative and fundamental policy of the New Medical Reform, and was officially implemented nationwide since 2016. Over the years, different practice models of FDCS have been explored and implemented in several large and well-developed cities, such as the “first contact care” model in Beijing, “1 (family doctor) + 1 (district hospital) + 1 (municipal hospital)” model in Shanghai, and the “Medication-Rehabilitation-Nursing Service” model in Hangzhou, while the FDCS system in other relatively underdeveloped areas has not yet been well established.

The FDCS system has been carried out in over 50 countries and regions worldwide, including Germany, (2) the United Kingdom, (3) the United States, (4) and Australia (5). Unlike European and American countries where the FDCS is mandatory, in China it is voluntary and free for local residents to sign with family doctors, with a contract period lasting for only 1 year. Although the Chinese medical care system has greatly improved medical access by reducing financial barriers and expanding community hospitals and family doctors, the FDCS system is still in an early stage and its potential is yet to be realized. Surveys reported a relatively low signing rate or utilization in China, which could be due to a shortage of family doctors and absence of supporting policies. The signing rate of FDCS ranged from 21.5 to 39.1% (6–8) and the utilization rate was from 23.8 to 34.3% according to different regional surveys with very small sample size (9, 10).

Investigating factors associated with the utilization of FDCS, rather than the willingness to renew contract, may help improve the generalizability and utilization of FDCS (11). Furthermore, establishing a predictive model for FDCS utilization may help improve the implementation of FDCS in other developing countries. To our knowledge, no studies have established a model with social demographics and environmental indicators to predict the utilization of FDCS. In addition, there were only small sample-size studies in China to assess the impact of social demographics on the service coverage and health management services. To address these knowledge gaps, we derived and validated a prediction model to predict the utilization of FDCS, leveraging on a national study with the largest sample size in China.

## Methods

### Study Design and Population

Two-stage cluster random sampling design was used to create a nationally representative sample of community people in China from March 2019 to April 2019. Surveys included 31 provinces [including four direct-controlled municipalities (Beijing, Tianjin, Shanghai, and Chongqing)] across China. In the first stage, we stratified China into six groups of economic-geographical regions (eastern rural, central rural, western rural, eastern urban, central urban, and western urban). We used these groups because the community health center volumes and clinical capacities differed significantly among the six official economic-geographical regions of mainland China. In each province, we selected primary health institutions (community health centers or township health centers) in proportion to the local population size and the total number of districts/counties.

In the second stage, we used systematic random sampling procedures to select permanent residents (defined as those who had lived in the district for more than 6 months, regardless of registration type and location) from the local health center database of the sampled community centers. In total, 165,308 subjects from 31 provinces in mainland China participated in the present study. The sample size and locations of these areas are shown in Supplementary Figure S1.

### Data Collection

Adults (aged >18 years) who were able to read and complete the questionnaire independently were recruited to the study. Only one member of a family (generally the primary income earner or the main service user) in contract with a family doctor was recruited in this study. We excluded residents with reading problems or with documented psychiatric disorders. An electronic questionnaire was used to collect the residents' socio-demographic characteristics, self-evaluation of health status, lifestyle, medical insurance, medical history, access of health services, and the status of FDCS. We used the question “Currently, have you signed with a family doctor and received healthcare services from them?” to define the status of FDCS.

All procedures performed in this study were in accordance with the 1964 Helsinki declaration and its later amendments. Patients voluntarily participated in the study. Before filling out the questionnaire, all participants provided informed consent on the first page of the questionnaire. Our study was approved by the Ethics Committee of Tongji Medical College institutional review board, Huazhong University of Science and Technology, Wuhan, China. All investigations were performed in accordance with the institutional guidelines. All data were anonymized and handled confidentially.

### Sample Size Consideration

The sample size was *post-hoc* calculated based on the rule of thumb recommended by Peduzzi et al. and Steyerberg et al., namely, events per variable (EPV) being 10 or greater under this setting of multiple regression model (12, 13). We considered around 15 significant clinical factors in developing a model. This would have required a minimum sample size of 150 participants who had events to predict the outcome of FDCS. We included 7,874 subjects in training dataset and 3,375 subjects in validation dataset.

### Statistical Analysis

We constructed multivariate prediction models that followed the TRIPOD statement (Transparent Reporting of a Multivariable Prediction Model for Individual Prognosis or Diagnosis) (14). Missing data were not imputed because <1% of data for any predictor variable were missing from the dataset. Descriptive statistics for categorical variables were reported as number (percentage) and were compared using the Pearson χ2 test or Fisher's exact test, as appropriate. Variables significant at the 0.1 level in univariate analyses were considered. Collinearity diagnosis was performed using the Spearman correlation and Belsley collinearity test. We tested for collinearity of all covariates and eliminated covariates that showed collinearity with other variables.

Stepwise multi-variable logistic regression analysis with backward selection was performed. Covariates included geographical regions, residence location, age, gender, ethnicity, population property, self-reported household income, education, marital status, occupation type, medical insurance, self-reported health status, smoking and drinking habits, self-reported exercise situation, chronic disease, walking distance from nearest community center, and illness in the last 2 weeks. Besides, we also applied the adaptive least absolute shrinkage and selection operator (LASSO) penalized regression models to select the most important variables to avoid overfitting. In the adaptive LASSO penalized regression model, 10-fold cross-validation was applied to determine the minimum value of λ, with no penalty on the same covariates of the multivariable logistic model. Odds ratios (OR) and 95% confidence intervals (CI) were obtained. Discrimination was evaluated using the area under the curve (AUC) derived from the conventional receiver operating characteristic curves (ROC). AUC as a measure of classification accuracy was further compared among the two models using the nonparametric approach of DeLong and Clarke-Pearson (15). Finally, a nomogram was obtained from a multivariable model and included 11 variables. The points of each predictor in the nomogram were first determined by drawing a vertical line from the factor to the point axis. The sum of all the points from all the predictors was then used to generate the total points. Next, validation and calibration of the best-fit model and nomogram were performed using bootstrapping methods (16). The bootstrap method was used with 1,000 resamples, and the bootstrap-corrected AUC and 95% CI were reported. Hosmer–Lemeshow test was used to assess the calibration plots of the nomogram (17). The validity of the model was verified in the validation dataset with respect to discrimination and calibration. All tests were two-sided with an alpha level of 0.05.

The statistical analysis was carried out using the programs SAS software (SAS v9.4; SAS Institute, NC, USA) and R v3.6.0 (R Foundation for Statistical Computing, Vienna, Austria).

## Results

### National Cross-Section Data Description

**Figure 1:**
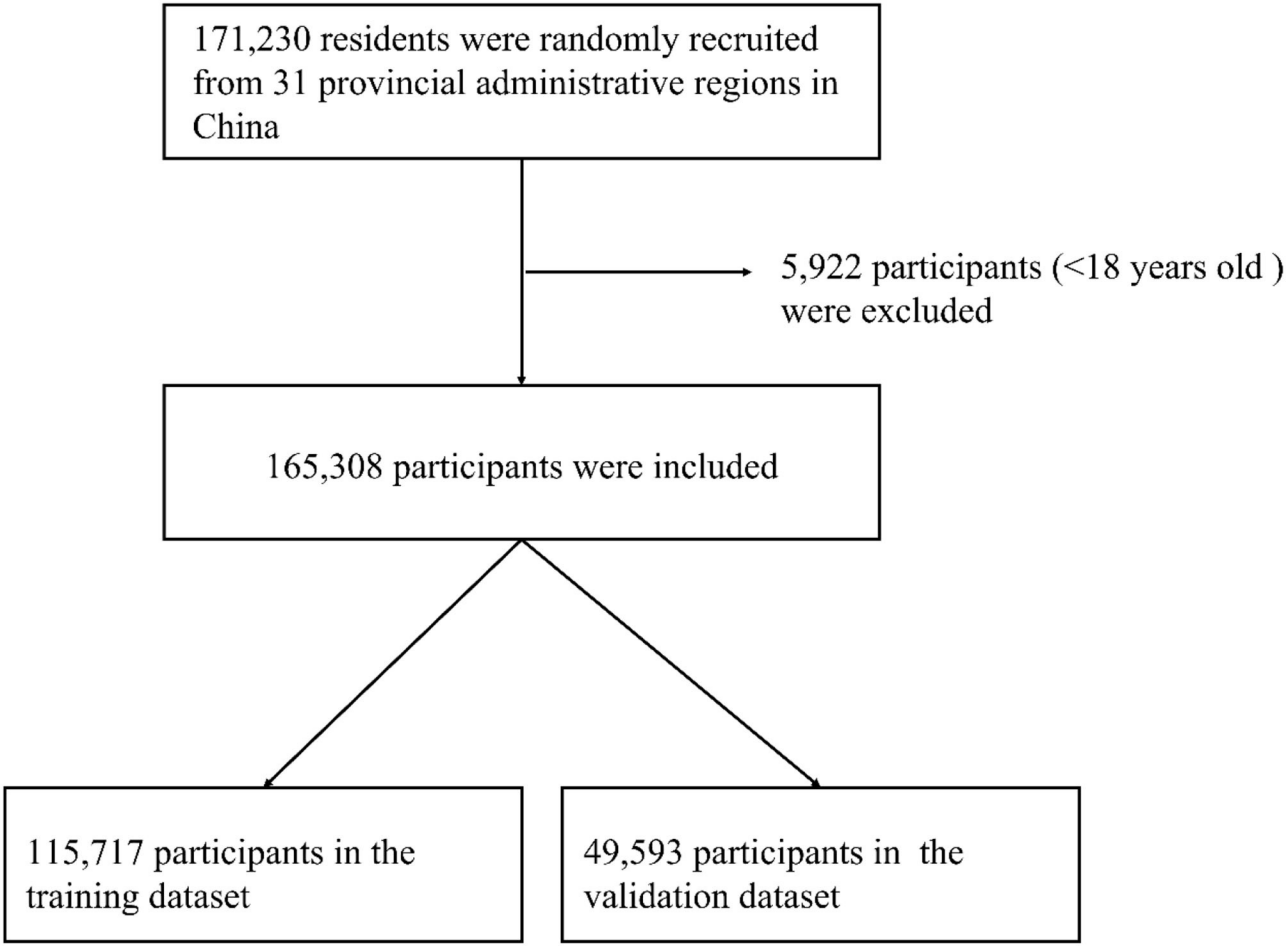
The flow chart for the sampling in this study: 31 provincial administrative regions, China.

A total of 115,715 subjects were randomly assigned to the training group (70% of the total cases) to develop prediction models. The rest of 49,593 subjects (30% of the total cases) were assigned to the validation groups to assess the model performance (Figure 1).

Overall, the median age of the participants was 28 years. The total FDCS utilization rate was 6.8%. Descriptive characteristics for the participants are summarized in Table 1. Residence location, age, gender, population property, self-reported household income, education, occupation type, medical insurance, self-reported health status, smoking and drinking habits, self-reported exercise situation, chronic disease, walking distance from nearest community center, illness in last 2 weeks, and treatment for illness in last 2 weeks were different between the FDCS and the no-FDCS groups in the development dataset in univariate regression analysis (*p* < 0.05), which was also observed in the validation dataset (Table 1).

**Table 1:** Baseline characteristics of the derivation dataset and validation dataset.

		**Derivation dataset, *n* (%)**				**Validation dataset, *n* (%)**			
		**Total**	**No FDCS**	**FDCS**	***p*-value**	**Total**	**No FDCS**	**FDCS**	***p*-value**
Total		115,717 (100.0)	107,824 (93.2)	7,893 (6.8)		49,593 (100.0)	46,234 (93.2)	3,357 (6.8)	
Geographical regions	Western urban	40,340 (69.9)	37,444 (92.8)	2,896 (7.2)	<0.001	17,361 (30.1)	16,118 (92.8)	1,243 (7.2)	0.001
	Central urban	15,171 (70.0)	14,143 (93.2)	1,028 (6.8)		6,511 (30.0)	6,065 (93.2)	446 (6.8)	
	Eastern urban	13,963 (69.8)	13,048 (93.4)	915 (6.6)		6,029 (30.2)	5,621 (93.2)	408 (6.8)	
	Western rural	28,240 (70.2)	26,482 (93.8)	1758 (6.2)		11,990 (29.8)	11,284 (94.1)	706 (5.9)	
	Central rural	8,553 (69.7)	7,939 (92.8)	614 (7.2)		3,711 (30.3)	3,448 (92.9)	263 (7.1)	
	Eastern rural	9,450 (70.3)	8,768 (92.8)	682 (7.2)		3,989 (29.7)	3,698 (92.7)	291 (7.3)	
Age, years	18–44	92,512 (70.0)	86,212 (93.2)	6,300 (6.8)	<0.001	39,593 (30.0)	36,917 (93.2)	2,676 (6.8)	0.007
	45–64	20,490 (69.9)	19,061 (93.0)	1,429 (7.0)		8,836 (30.1)	8,223 (93.1)	613 (6.9)	
	65–79	2,397 (70.6)	2,275 (94.9)	122 (5.1)		999 (29.4)	950 (95.1)	49 (4.9)	
	80-	318 (66.1)	276 (86.8)	42 (13.2)		163 (33.9)	144 (88.3)	19 (11.7)	
Gender	Female	62,345 (69.8)	58,696 (94.1)	3,649 (5.9)	<0.001	26,926 (30.2)	25,286 (93.9)	1,640 (6.1)	<0.001
	Male	53,372 (70.2)	49,128 (92.0)	4,244 (8.0)		22,665 (29.8)	20,948 (92.4)	1,717 (7.6)	
Ethnic	Minority	10,775 (69.8)	10,026 (93.0)	749 (7.0)	0.573	4,652 (30.2)	4,354 (93.6)	298 (6.4)	0.300
	Han	10,4942 (70.0)	97,798 (93.2)	7,144 (6.8)		44,939 (30.0)	41,880 (93.2)	3,059 (6.8)	
Population property	Migrant	38,069 (70.0)	36,123 (94.9)	1,946 (5.1)	<0.001	16,298 (30.0)	15,414 (94.6)	884 (5.4)	<0.001
	Permanent resident	77,648 (70.0)	71,701 (92.3)	5,947 (7.7)		33,293 (30.0)	30,820 (92.6)	2,473 (7.4)	
Self-reported household income	Low	24,817 (69.8)	23,347 (94.1)	1,470 (5.9)	<0.001	10,745 (30.2)	10,159 (94.5)	586 (5.5)	<0.001
	Middle	63,146 (70.0)	59,629 (94.4)	3,517 (5.6)		27,105 (30.0)	25,543 (94.2)	1,562 (5.8)	
	High	27,754 (70.3)	24,848 (89.5)	2,906 (10.5)		11,741 (29.7)	10,532 (89.7)	1,209 (10.3)	
Education	Illiteracy /primary / middle school	37,659 (69.9)	35,125 (93.3)	2,534 (6.7)	<0.001	16,179 (30.1)	15,128 (93.5)	1,051 (6.5)	<0.001
	Completion of high schoo	15,776 (70.5)	14,804 (93.8)	972 (6.2)		6,586 (29.5)	6,165 (93.6)	421 (6.4)	
	College	56,589 (69.9)	52,838 (93.4)	3751 (6.6)		24,400 (30.1)	22,788 (93.4)	1,612 (6.6)	
	Master or above	5,693 (70.1)	5,057 (88.8)	636 (11.2)		2,426 (29.9)	2,153 (88.7)	273 (11.3)	
Marital status	Married/others	70,181 (70.0)	65,458 (93.3)	4,723 (6.7)	0.127	30,022 (30.0)	28,039 (93.4)	1,983 (6.6)	0.071
	single	45,536 (69.9)	42,366 (93.0)	3,170 (7.0)		19,569 (30.1)	18,195 (93.0)	1,374 (7.0)	
Occupation type	Full time job	44,066 (70.2)	67,043 (93.6)	4,608 (6.4)	<0.001	18,671 (29.8)	28,971 (93.7)	1,949 (6.3)	<0.001
	Part time job/retired/other	71,651 (69.9)	40,781 (92.5)	3,285 (7.5)		30,920 (30.1)	17,263 (92.5)	1,408 (7.5)	
Medical insurance	No	9,498 (70.3)	9,030 (95.1)	468 (4.9)	<0.001	4,020 (29.7)	3,828 (95.2)	192 (4.8)	<0.001
	Basic medical insurance for urban and rural residents	62,158 (69.9)	58,625 (94.3)	3,533 (5.7)		26,753 (30.1)	25,245 (94.4)	1,508 (5.6)	
	Public medical care	4,257 (69.9)	3,997 (93.9)	260 (6.1)		1,829 (30.1)	1,704 (93.2)	125 (6.8)	
	business insurance	6,452 (70.5)	5,927 (91.9)	525 (8.1)		2,701 (29.5)	2,484 (92.0)	217 (8.0)	
	Urban employee medical insurance	33,352 (70.0)	30,245 (90.7)	3,107 (9.3)		14,288 (30.0)	12,973 (90.8)	1,315 (9.2)	
Self-reported health status	Poor	6,354 (69.5)	6,036 (95.0)	318 (5.0)	<0.001	2,782 (30.5)	2,651 (95.3)	131 (4.7)	<0.001
	General	43,762 (69.9)	41,351 (94.5)	2,411 (5.5)		18,806 (30.1)	17,775 (94.5)	1,031 (5.5)	
	Good	65,601 (70.1)	60,437 (92.1)	5,164 (7.9)		28,003 (29.9)	25,808 (92.2)	2,195 (7.8)	
Cigarette, per day	No smoking	90,612 (70.0)	84,366 (93.1)	6,246 (6.9)	<0.001	38,837 (30.0)	36,166 (93.1)	2,671 (6.9)	0.134
	<20	23,148 (70.0)	21,673 (93.6)	1,475 (6.4)		9,936 (30.0)	9,308 (93.7)	628 (6.3)	
	≥20	1,957 (70.5)	1,785 (91.2)	172 (8.8)		818 (29.5)	760 (92.9)	58 (7.1)	
Alcohol drinking	Yes	25,105 (70.0)	23,458 (93.4)	1,647 (6.6)	<0.001	10,754 (30.0)	10,068 (93.6)	686 (6.4)	<0.001
	No	90,612 (70.0)	84,366 (93.1)	6,246 (6.9)		38,837 (30.0)	36,166 (93.1)	2,671 (6.9)	
Self-reported exercise situation	Never	53,646 (70.2)	50,351 (93.9)	3,295 (6.1)	<0.001	22,770 (29.8)	21,370 (93.9)	1,400 (6.1)	<0.001
	1–2 per week	37,812 (69.8)	35,192 (93.1)	2620 (6.9)		16,384 (30.2)	15,285 (93.3)	1,099 (6.7)	
	3–5 per week	16,681 (69.9)	15,431 (92.5)	1250 (7.5)		7,175 (30.1)	6,628 (92.4)	547 (7.6)	
	≥6 per week	7,578 (69.9)	6,850 (90.4)	728 (9.6)		3,262 (30.1)	2,951 (90.5)	311 (9.5)	
Chronic disease	No	17,755 (70.3)	16,698 (94.0)	1,057 (6.0)	<0.001	7,500 (29.7)	7,059 (94.1)	441 (5.9)	0.001
	yes	97,962 (69.9)	91,126 (93.0)	6,836 (7.0)		42,091 (30.1)	39,175 (93.1)	2,916 (6.9)	
Illness last 2 week	No	92,552 (70.0)	87,555 (94.6)	4,997 (5.4)	<0.001	39,653 (30.0)	37,520 (94.6)	2,133 (5.4)	<0.001
	Yes	23,165 (70.0)	20,269 (87.5)	2,896 (12.5)		9,938 (30.0)	8,714 (87.7)	1,224 (12.3)	
Treatment while illness last 2 week	No illness	92,552 (70.0)	87,555 (94.6)	4,997 (5.4)	<0.001	39,653 (30.0)	37,520 (94.6)	2,133 (5.4)	<0.001
	Rest at home	7,866 (70.3)	6,321 (80.4)	1545 (19.6)		3,329 (29.7)	2,672 (80.3)	657 (19.7)	
	Bug drug privately	7,889 (69.4)	7,289 (92.4)	600 (7.6)		3,478 (30.6)	3,230 (92.9)	248 (7.1)	
	Visit hospital	4,019 (70.3)	3,526 (87.7)	493 (12.3)		1,698 (29.7)	1,517 (89.3)	181 (10.7)	
	Vist community center	3,391 (70.3)	3,133 (92.4)	258 (7.6)		1,433 (29.7)	1,295 (90.4)	138 (9.6)	
Walking distance from nearest Community center	≥30 min walking	22,113 (69.9)	21,211 (95.9)	902 (4.1)	<0.001	9,543 (30.1)	9,163 (96.0)	380 (4.0)	<0.001
	15–29 min walking	37,991 (70.1)	35,788 (94.2)	2,203 (5.8)		16,195 (29.9)	15,265 (94.3)	930 (5.7)	
	<15 min walking	55,613 (70.0)	50,825 (91.4)	4,788 (8.6)		23,853 (30.0)	21,806 (91.4)	2,047 (8.6)	

### Multivariable Logistic Regression Analysis for Predictors of FDCS

**Figure 2:**
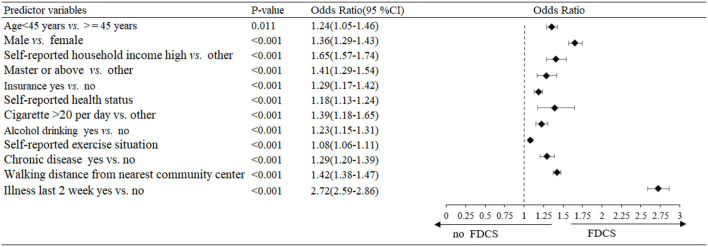
Multivariable logistic regression analysis of predictors for the family doctor contract service (FDCS) in the development dataset.

On the basis of initial screening, multivariable logistic regression analysis was performed including 12 variables (age <45 years, male, high self-reported household income, education level of master or above, having medical insurance, good self-reported health status, cigarettes >20 per day, alcohol drinking, more self-reported exercise situation, chronic disease, less walking distance from nearest community center, illness in last 2 week) that were statistically significant (P <0.05) in the univariate analysis. Subjects with above characteristics were more likely to utilize and receive FDCS service, with the ORs ranging from 1.08 (1.06–1.11) to 2.72 (2.59–2.86) (Figure 2).

In sensitivity analysis, an altered model that included category forms of scale insurance, self-reported health status, self-reported exercise situation, and walking distance from nearest community center showed similar findings as in the primary analysis (Supplementary Figure S2).

### Prediction Nomogram for Application

**Figure 3:**
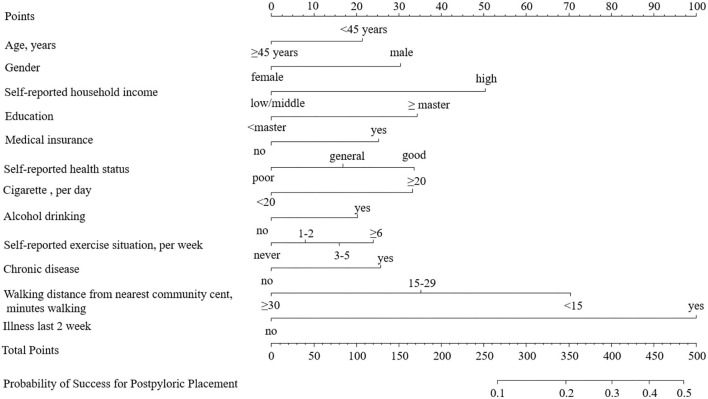
The nomogram for the family doctor contract service. The points of each predictor were firstly determined by drawing a vertical line from the factor to the point axis. The sum of all the points from all predictors was then used to generate the total points. By drawing a vertical line from the total point axis to the risk of RAS axis, the estimated probability of the family doctor contract service could be obtained.

**Figure 4:**
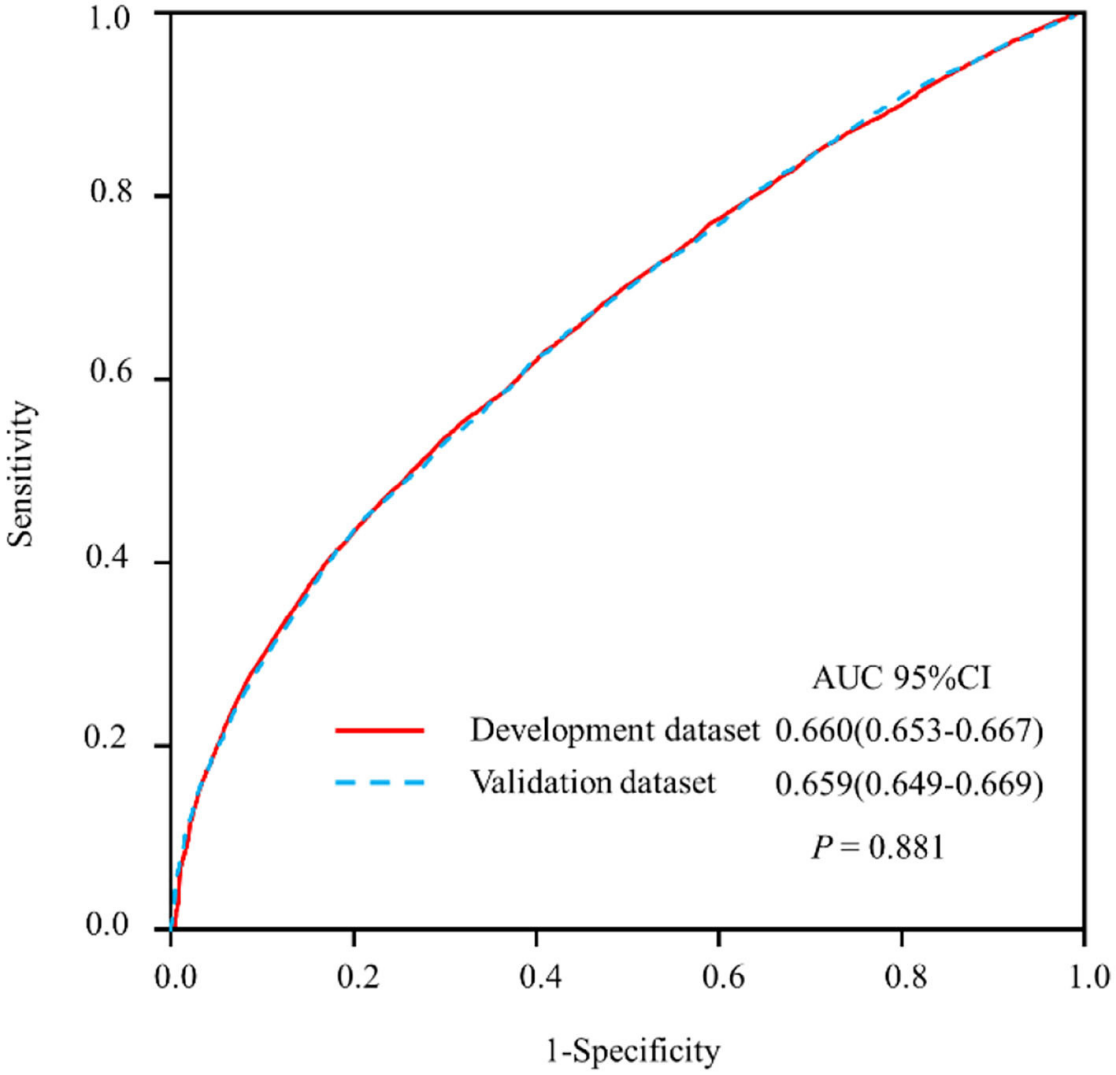
Receiver operating characteristic curve (ROC) analyses of predictors for the family doctor contract service in the development and validation datasets.

**Figure 5:**
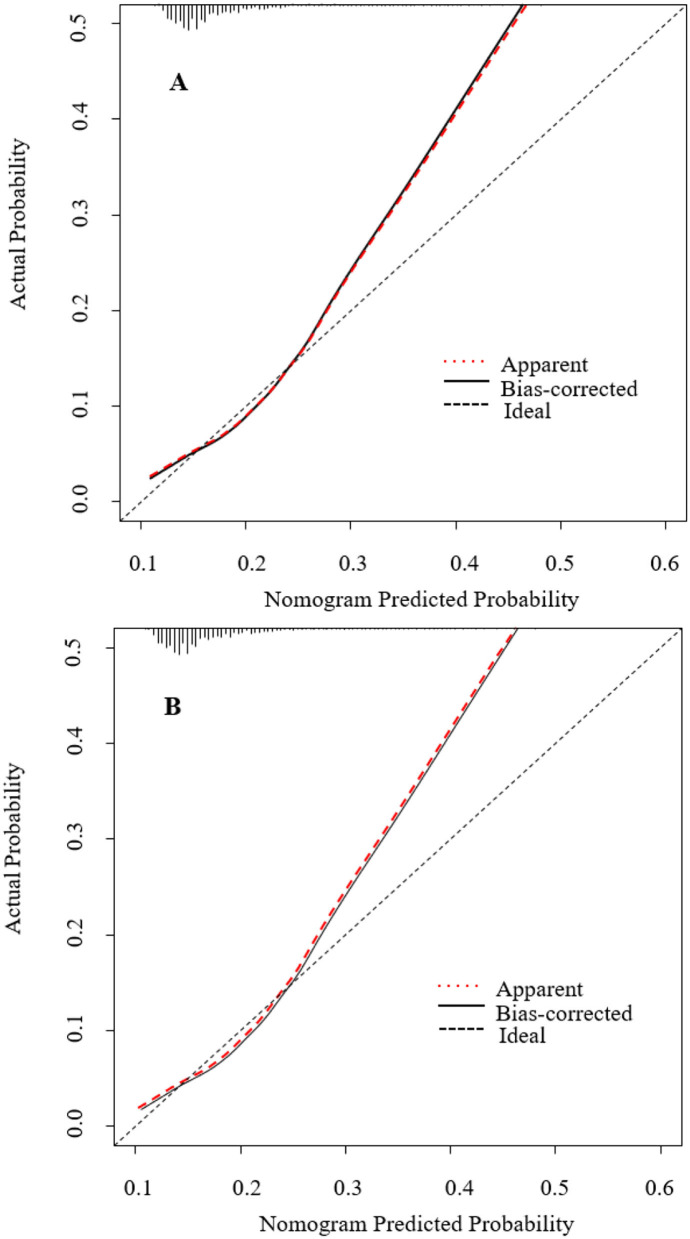
Calibration plot for nomogram in the **(A)** development dataset and **(B)** validation dataset. The 45° dashed line represents ideal predictions. The plot illustrates the accuracy of the multivariable model (“Apparent”) and the bootstrap model (“Bias-corrected”) for predicting willingness of family doctor. Locally weighted scatterplot smoothing was used to illustrate the relationships of the two models with the ideal line. Both plots are linear and agree well in low predicted probabilities ≤ 0.3, but the disagreement between the two plots grows with the predicted probability >0.3. The 0.9 quantile absolute errors of the predicted probability are 0.012 (development cohort) and 0.013 (validation cohort), respectively. The black dots illustrate the relationship between the predicted probability and observed probability of the nomogram for predicting family doctor contract service in the original data set.

The model that incorporated the identified independent predictors in the multivariable logistic analysis was completed and presented as the nomogram (Figure 3). ROC analyses of predictors for the utilization of FDCS in the development and validation cohort are shown in Figure 4. The AUC for the development and validation cohorts were 0.660 (0.653–0.667) and 0.659 (0.649–0.669), respectively, without significant difference (DeLong test, *p* = 0.881). The calibration curve for the probability of FDCS in the development and validation cohorts demonstrated good agreement between prediction and observation (Figures 5A,B). Both plots are slightly non-linear and agreed well in predicting a low utilization rate of FDCS <30%, and the disagreement between the two plots slightly grows with the predicted probability of success of utility of FDCS ≥30%.

## Discussion

In this national, population-based cross-sectional study, the utilization of FDCS can be predicted moderately based on age, male, self-reported household income, education, insurance, self-reported health status, cigarettes smoked per day, alcohol drinking, self-reported exercise situation, chronic disease, walking distance from nearest community center, and illness in the last 2 weeks. The model incorporates routinely available variables that are obtainable in daily primary practice. Public health workers can apply this handy tool to identify high-utility groups for individualized counseling or contract. Our simple nomogram is designed to facilitate the use of the model in primary healthcare practice.

In the present study, approximately 13% residents who contracted with and received healthcare services from family doctors were older than 80 years. Severe illness and the need for targeted, convenient, and continuous healthcare were the main drivers of FDCS among older adults (18). Compared with females and low household income, males and high-income participants were more likely to receive FDCS service. It could be due to their better understanding of social information and government politics among the residents with high social status (19). Educational level is directly related to the level of health status, as individuals with health illiteracy have difficulty understanding medical information and advice (20). In the present study, medical insurance and better self-reported health status were associated with higher FDCS utilization. The reason could be that this population had stronger economic capacity, better access to medical services, and paid more attention to their own health status. In addition, we found that residents who were more health-conscious chose a healthier lifestyle, including lesser consumption of cigarettes and alcohol. It is likely that these people had greater health awareness, healthier behavior, better education, and higher personal income (21).

In China, improving FDCS utilization was a key breakthrough in establishment of a tiered medical system of primary healthcare, diagnosis, and treatment. However, the utilization rate was found to be 6.8% in this study, which was lower than that in previous studies with regional design and very small sample size (utilization rate, 23.8–34.3%) (9, 10). This discrepancy could be attributed to multiple reasons, including limited educational level of family doctors, mutual-mistrust between doctor and patient, and lack of healthcare providers. The levels of education and qualification among primary healthcare professionals in China are relatively low. In 2018, 25% of family doctors had less than a junior medical college level of education (22, 23). Moreover, due to a shortage of qualified physicians, more than 20% of doctors practicing in community health centers were not licensed (23). As a result, patients were inclined to bypass primary healthcare institutions when they needed clinical care (32%), and 26% of patients responded that they distrusted community health centers (24). Besides, the shortage of FDCS teams has persisted, with 3.7 licensed physicians per one thousand people in urban China and 1.3 in rural areas, (25) which limits their functional response to the needs of the community (26).

To our knowledge, this study is the first attempt to establish a nomogram for predicting the utilization of FDCS in a nation-represented study. We developed the model using data from a national, high-quality, cross-sectional study. We evaluated predictors that were epidemiologically relevant and routinely available to primary healthcare providers, so that the model can be easily applied in primary healthcare practice. In addition, the data for external validation of nomogram was also from large, randomized, and multicenter data. A convenient and easy-to-use nomogram of the FDCS model allows for immediate use of the model to predict the utilization of FDCS.

Despite the strengths of this study, several limitations need to be acknowledged. First, the cross-sectional design of this study limits its ability to identify the causal relationships. Second, other factors that might impact FDCS use were not included in our survey, such as residents' psychological status, income, or expenditure, and the publicity of FDCS. Third, since the proportion of participants aged 65 years and older was low in this study, the implication of our findings for the elder population might be limited. Therefore, our results need further validation in a multi-institution study with larger samples. Additionally, the performance of the nomogram is only slightly higher than 0.65. Considering that all predictors are easily accessible in primary healthcare with low cost, the moderate diagnostic performance of the prediction nomogram in our study should be acceptable. Although it provides insights into novel domain discovery, the nomogram model might be affected by factors such as sample quality variations and variables such as the income or expenditure information, and further validations are needed. Finally, since the present study was conducted only in Chinese population, these results might not be extrapolated to other countries.

## Conclusions

A prediction nomogram that incorporates 12 easily accessible variables in daily practice of community primary healthcare can be used to predict residents' utilization of FDCS. These findings provide an important insight for the government to guide the focus of primary healthcare at individual, community, and national levels. To further improve the performance of the predictive model, more clinically important variables need to be considered.

## Data Availability Statement

The raw data supporting the conclusions of this article will be made available by the authors, without undue reservation.

## Ethics Statement

The studies involving human participants were reviewed and approved by the Ethics Committee of Tongji Medical College Institutional Review Board. The patients/participants provided their written informed consent to participate in this study.

## Author Contributions

ZL and YF conceived and designed the research. CW and YG performed the research and collected data. ZN analyzed the data and wrote the manuscript. CC and GC revised the manuscript critically for important intellectual content. All authors read and approved the final manuscript.

## Funding

This study was supported by grants from the Natural Science Foundation of Guangdong Province (2020A1515010743), the National Social Science Foundation of China (18ZDA085), the Science and Technology Plan Program of Guangzhou (No. 201803040012), and the Key Area R&D Program of Guangdong Province (No. 2019B020227005).

## Conflict of Interest

The authors declare that the research was conducted in the absence of any commercial or financial relationships that could be construed as a potential conflict of interest.

## Publisher's Note

All claims expressed in this article are solely those of the authors and do not necessarily represent those of their affiliated organizations, or those of the publisher, the editors and the reviewers. Any product that may be evaluated in this article, or claim that may be made by its manufacturer, is not guaranteed or endorsed by the publisher.

## Supplementary Material

Supplementary Figure S1 The geographic distribution of participants in the survey. Figure legend: The sample population of this study was from all over China, except Taiwan, Hong Kong, and Macau. This ArcGIS image was marked with the city as the unit, and the darker the color, the more people were sampled.

Supplementary Figure S2 Multivariate logistic regression analysis of predictors for the family doctor contract service in the development dataset with category-scale variables.

## Abbreviations

AUC: Area under the receiver operating characteristic curve
CI: Confidence intervals
FDCS: Family doctor contract service
OR: Odds ratio
ROC: Receiver operating characteristic curves.
