# Supplementary figures and images for: Development and Validation of a Model to Predict the Contract Service of Family Doctor: A National Survey in China
Source: Front Public Health. 2022 Apr 25;10:750722. doi: 10.3389/fpubh.2022.750722 (PMC9082311; doi:10.3389/fpubh.2022.750722)

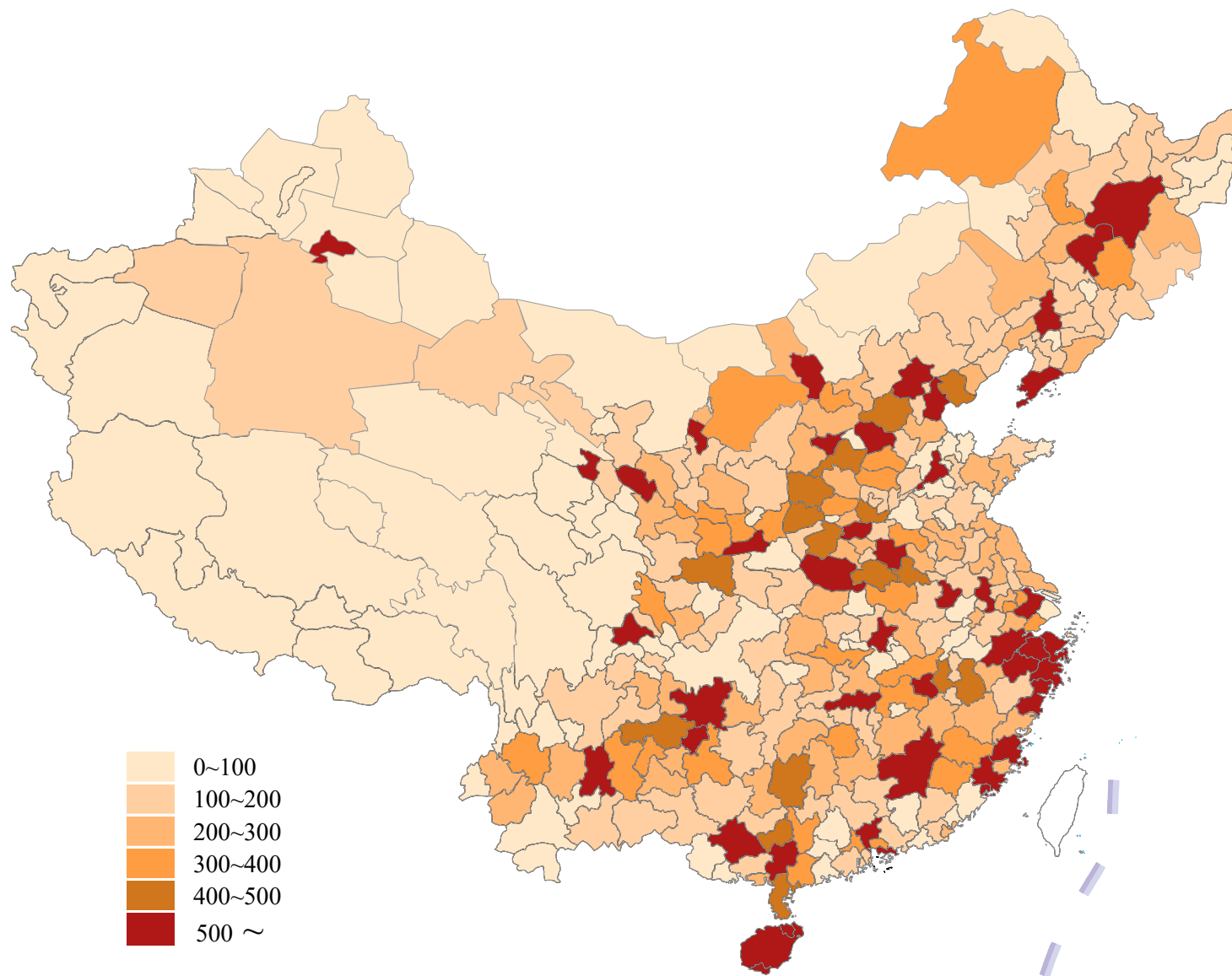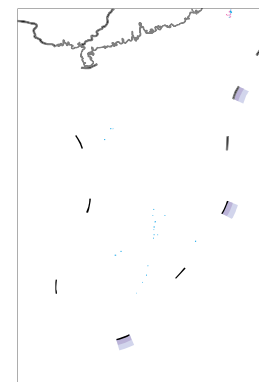

Supplement: Supplementary Figure S1 — The geographic distribution of participants in the survey. Figure legend: The sample population of this study was from all over China, except Taiwan, Hong Kong, and Macau. This ArcGIS image was marked with the city as the unit, and the darker the color, the more people were sampled. [file Data_Sheet_1.PDF]

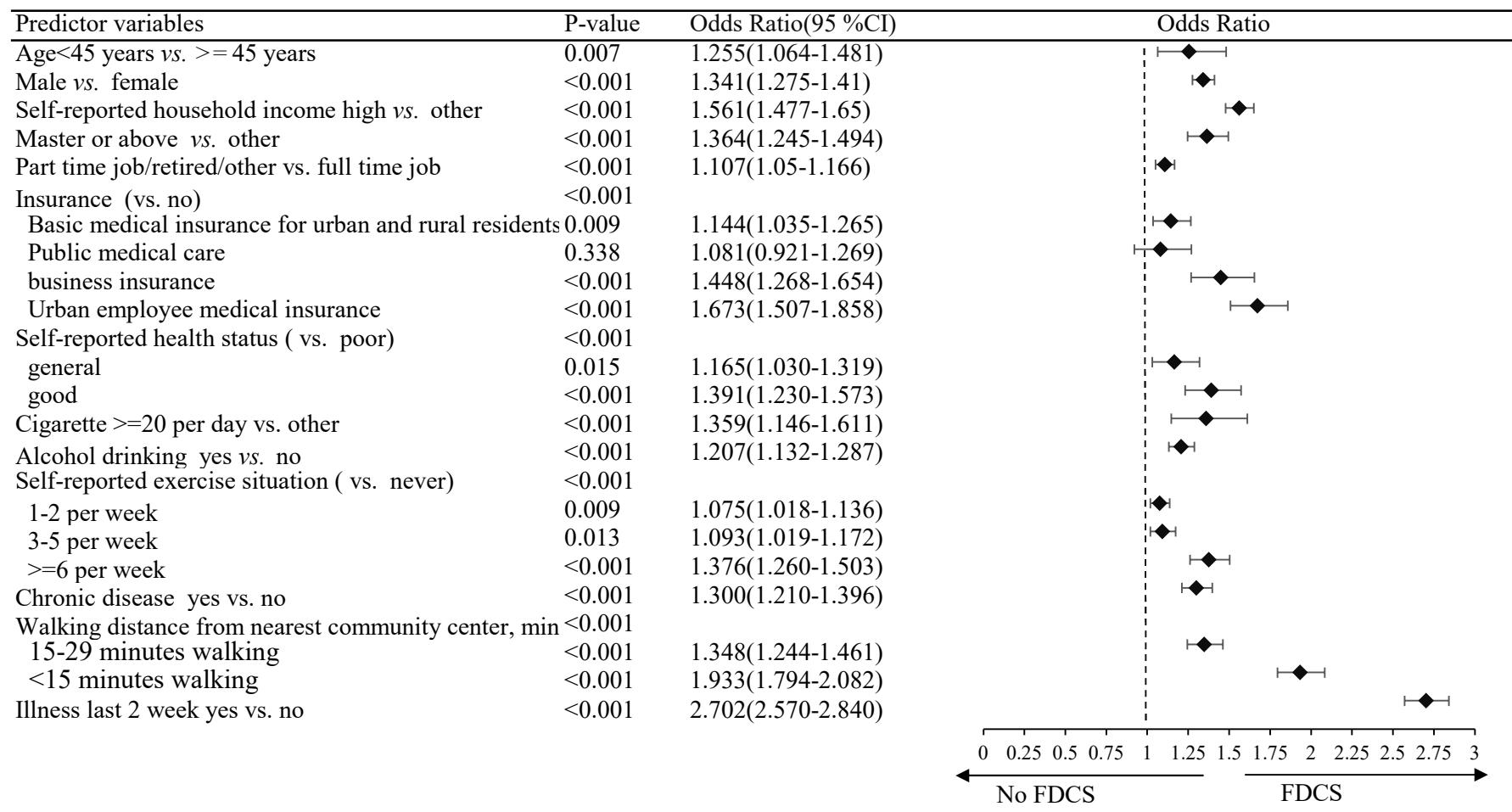

Supplement: Supplementary Figure S2 — Multivariate logistic regression analysis of predictors for the family doctor contract service in the development dataset with category-scale variables. [file Data_Sheet_2.PDF]
